# Integrated Metabolomics and Volatolomics for Comparative Evaluation of Fermented Soy Products

**DOI:** 10.3390/foods10112516

**Published:** 2021-10-20

**Authors:** Sang-Hee Lee, Sunmin Lee, Seung-Hwa Lee, Hae-Jin Kim, Digar Singh, Choong-Hwan Lee

**Affiliations:** 1 Department of Bioscience and Biotechnology, Konkuk University, Seoul 05029, Korea; tkdgml823@hanmail.net (S.-H.L.); duly123@naver.com (S.L.); singhdigar@gmail.com (D.S.); 2 Experiment Research Institute, National Agricultural Products Quality Management Service, Gimcheon-si 39660, Korea; shlee96@korea.kr (S.-H.L.); asarela00@korea.kr (H.-J.K.)

**Keywords:** fermented soy product, metabolomics, volatolomics, metabolic pathway

## Abstract

Though varying metabolomes are believed to influence distinctive characteristics of different soy foods, an in-depth, comprehensive analysis of both soluble and volatile metabolites is largely unreported. The metabolite profiles of different soy products, including cheonggukjang, meju, doenjang, and raw soybean, were characterized using LC-MS (liquid chromatography–mass spectrometry), GC-MS (gas chromatography–mass spectrometry), and headspace solid-phase microextraction (HS-SPME) GC-MS. Principal component analysis (PCA) showed that the datasets for the cheonggukjang, meju, and doenjang extracts were distinguished from the non-fermented soybean across PC1, while those for cheonggukjang and doenjang were separated across PC2. Volatile organic compound (VOC) profiles were clearly distinct among doenjang and soybean, cheonggukjang, and meju samples. Notably, the relative contents of the isoflavone glycosides and DDMP (2,3-dihydro-2,5-dihydroxy-6-methyl-4H-pyran-4-one) conjugated soyasaponins were higher in soybean and cheonggukjang, compared to doenjang, while the isoflavone aglycones, non-DDMP conjugated soyasaponins, and amino acids were significantly higher in doenjang. Most VOCs, including the sulfur containing compounds aldehydes, esters, and furans, were relatively abundant in doenjang. However, pyrazines, 3-methylbutanoic acid, maltol, and methoxyphenol were higher in cheonggukjang, which contributed to the characteristic aroma of soy foods. We believe that this study provides the fundamental insights on soy food metabolomes, which determine their nutritional, functional, organoleptic, and aroma characteristics.

## 1. Introduction

Fermentation is a metabolic bioprocess that causes biochemical changes in the organic substrate, through the action of microbial enzymes [1]. Fermentation is considered a customarily important method of food processing, as it adds superior taste, flavor, and nutrition to the end-product, as well as enhanced shelf life, compared to raw materials [2]. First cultivated in East Asia, thousands of years ago, soybean is an important, nutrient-rich crop, but the presence of anti-nutritive toxic substances may limit its nutritional level [3]. Thus, the fermentation approach was used to overcome these limitations and improve the quality of soybeans, such as the desired taste, aroma, and health effects.

**Figure 1:**
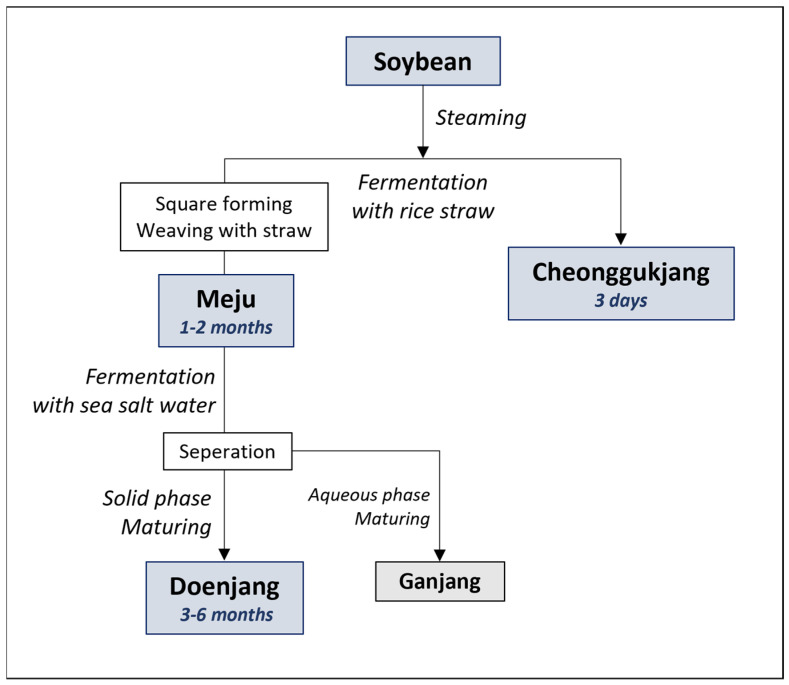
Manufacturing steps during the fermentative manufacturing of cheonggukjang, meju, and doenjang from raw soybean.

Depending on the fermentation process, various fermented foods are produced, such as cheonggukjang (fermented soybean), meju (fermented soybean block), doenjang (fermented soybean paste), ganjang (fermented soy sauce), and gochujang (fermented hot-pepper paste) [4]. Among them, the cheonggukjang, prepared traditionally, is made by steaming soybeans for several days with rice straw, and meju is made from boiled beans, wrapped in rice straw and fermented for several months. On the other hand, doenjang is made by fermenting the meju for an extended duration, ranging 3 months to 3 years, and separating the liquid layer as ganjang (Figure 1) [5]. Different fermented food products have varying flavors and nutritional functions, owing to the varying substrate processing and fermentation methods. However, the exact factors affecting the characteristics of each product are not precisely known.

Metabolite profiling has been used in previous studies to assess the metabolomes of different foods and biological systems, using a variety of hyphenated mass spectrometry (MS)-based platforms [4,6]. Untargeted metabolomic studies, involving soybean fermented foods, can unravel the biochemical aspects of associated nutritional and functional activities and may further reveal the biomarkers for their quality standardization.

The composition of volatile organic compounds, part of secondary metabolites in food, affects the taste, aroma, and properties, and, especially in fermented foods, it is considered an important criterion of consumer acceptability and require comprehensive analysis. SPME (solid phase microextraction) is a simple, solvent-free, and reliable microextraction technique to extract volatile compounds from solids, liquids, or even gasses [7,8]. It is considered a highly reproducible method and has been employed in food aroma and perfumery studies. This can detect volatile compounds that are highly unstable and difficult to detect in solvent-based extraction methods. Therefore, a combination of different spectroscopic platforms, such as GC–MS, LC–MS, and SPME–GC–MS, could detect broader classes of metabolites and volatile compounds.

A recent study described the effect of fermentation methods on changes in total metabolite content by comparing various soy fermented products [9]. However, soybean differs in nutritional content and characteristics, depending on cultured area and cultivar. This can be a biased factor when comparing the differences in metabolite content by the fermentation method. Within our literature search, the comparison of volatile compounds for different soy fermented foods has not yet been carried out. Thus, comparison of primary and secondary metabolites, as well as the volatile organic compounds (VOCs) for different commercially available fermented soy products, including cheonggukjang, meju, and doenjang, which were produced traditionally, was performed. Additionally, soybean, which is used as raw material in most fermented soy foods, was analyzed.

## 2. Materials and Methods

### 2.1. Chemicals and Reagents

HPLC-grade water, acetonitrile, and methanol were purchased from Fisher Scientific (Pittsburgh, PA, USA). Diethylene glycol was obtained from Junsei Chemical Co., Ltd. (Tokyo, Japan). Formic acid, methoxyamine hydrochloride, pyridine, potassium persulfate, N-methyl-N-(trimethylsilyl) trifluoroacetamide (MSTFA), 2,2′-azino-bis (3-ethylbenzothiazoline-6-sulfonic acid), diammonium salt (ABTS), 6-hydroxy-2,5,7,8-tetramethylchromane-2-carboxylic acid (Trolox), sodium acetate, acetic acid, 2,4,6-Tris (2-pyridyl)-s-triazine (TPTZ), hydrochloric acid (HCl), iron (III) chloride hexahydrate, sodium carbonate, Folin–Ciocalteu’s phenol, sodium hydroxide, gallic acid, and naringin were obtained from Sigma-Aldrich (St. Louis, MO, USA).

### 2.2. Sample Information

Twenty samples, (5 soybean and 15 soybean fermented foods (5 cheonggukjang, 5 meju, and 5 doenjang)) were purchased from the traditional manufacturers (Supplementary Materials Table S1). Five different types of soybeans, cheonggukjang, meju, and doenjang were obtained from each of the five manufacturers, in order to obtain soybean fermented foods made from the same soybeans. All the samples were stored at −20 °C, until analysis. Sample information and manufacturing steps are shown in Table S1 and Figure 1. The fermentation period was estimated based on a previous study [5].

### 2.3. Sample Extraction

#### 2.3.1. Extraction and Derivatization for Metabolite Profiling

Sample extraction was carried out, following the methodology used by Lee et al. [10], with some modifications. Approximately 100 mg of sample powder was extracted with 80% aqueous methanol (100 mg/mL), using a mixer mill (Retsch GmbH and Co, Haan, Germany) for 10 min at 30 Hz/s, after sonication for 5 min. The sample mixtures were then centrifuged at 8000× *g* for 10 min at 4 °C. The supernatant was separated, following the same procedure, repeated twice. All acquired samples were filtered through a 0.2 μm syringe filter, then dried using a speed vacuum concentrator (Biotron, Seoul, Korea). Because of the significantly different extraction yields, owing to the different physicochemical characteristics of samples, all extracts were normalized based on weight of samples (Table S2). The collected samples were resuspended in 80% methanol prior to the LC-MS and GC-MS procedures.

For the GC-MS analysis, sample extracts were added with methoxyamine hydrochloride (20 mg/mL) in pyridine and oximated at 30 °C for 90 min. Then, the oximated samples were silylated with MSTFA at 37 °C for 30 min. We maintained three replicates for fermented food sources and three analytical replicates.

#### 2.3.2. Extraction of Headspace Volatile Organic Compounds

Extraction of volatile compounds was carried out by modifying the method by Jo et al. [11]. Sample (2.5 g) was mixed with 2.75 mL of water, 0.75 g of NaCl, and 10 µL of linalool (2500 ppm in distilled water), an internal standard. The sample mixture was added in the 20 mL amber SPME vials with a silicon/teflon septum (Supelco, Bellefonte, PA, USA). The mixtures were maintained at 60 °C for 30 min, and SPME fiber (1 cm), coated with 75 µm carboxen/polydimethylsiloxane/divinylbenzene (CAR/PDMS/DVB), was exposed at 40 °C for 30 min to the headspace. The fiber had a desorption procedure at 230 °C for 5 min in a GC injector, and the analysis was performed in splitless mode.

### 2.4. Instrumentations

An Agilent 7890A GC system (Agilent Technologies, Palo Alto, CA, USA), comprising an Agilent 7693 autosampler, coupled with a TOF Pegasus III mass spectrometer (LECO, St. Joseph, MI, USA), was used in gas chromatography/time-of-flight mass spectrometry (GC–TOF-MS) analysis. An RTX-5MS column (30 m × 0.25 mm, 0.25 μm particle size; Restek Corp., Bellefonte, PA, USA) was used, with a flow of 1.5 mL/min of helium. Sample was injected with split ratio of 10:1. The oven temperatures, maintained at 75 °C for 2 min, increased to 300 °C at 15 °C/min, and then were maintained for 3 min. Details of the analysis and instrument operation conditions were adopted from the method suggested by Jung et al. [12].

Ultra-high performance liquid chromatography linear trap quadrupole tandem mass spectrometry (UHPLC-LTQ-orbitrap-MS/MS) was performed on an Vanquish Binary pump H system (Thermo Fisher Scientific, Waltham, MA, USA), equipped with auto-sampler. The column was an Phenomenex KINETEX^®^ C18 column (100 mm × 2.1 mm, 1.7 µm particle size; Torrance, CA, USA), and the injection volume was 5 μL. The column temperature was set to 40 °C, and the flow rate was 0.3 mL/min. The 0.1% (*v*/*v*) of formic acid in water (mobile phase A) and 0.1% (*v*/*v*) of formic acid in acetonitrile (mobile phase B) were used, respectively. The MS data were collected in the range of 100–2000 *m*/*z* using an ion trap mass spectrometer (Thermo Fisher Scientific, Waltham, MA, USA). The analytical program, applied for the sample, was adopted from the method described by Kwon et al. [13].

Headspace solid-phase microextraction-gas chromatography–mass spectrometry (HS-SPME/GC-TOF-MS) analysis was conducted. DB-FFAP column (30 m length × 0.25 mm internal diameter × 0.25 µm film thickness, J&W Scientific, Folsom, CA, USA) was used to separate a sample analyte. The oven temperature was initially set at 40 °C for 6 min, increased to 200 °C at a rate of 4 °C/min, and then maintained at 200 °C for 5 min. The flow rate of a carrier gas, helium, was 0.8 mL/min. A mass scan range of 50–400 *m*/*z* and 70 eV of ionization energy were set.

### 2.5. Data Processing and Multivariate Statistical Analysis

GC-TOF-MS and HS-SPME/GC-TOF-MS raw data files were converted to a network common data form (NetCDF) (*.cdf) with the ChromaTOF software (LECO, St. Joseph, MI, USA). The UHPLC-LTQ-Orbitrap-MS raw data were acquired and converted into netCDF (*.cdf) using Xcalibur software (version 2.1, Thermo Fisher Scientific). Both CDF data were processed with the MetAlign software (http://www.metalign.nl, (accessed on 16 September 2021)) for data alignment, in accordance with peak mass (*m*/*z*) and retention time (min). Multivariate statistical analysis was processed using SIMCA-P+ 12.0 software (Umetrics; Umeå, Sweden). Principal component analysis (PCA) and partial least squares discriminant analysis (PLS-DA) were used to compare metabolic differences of samples. The significantly discriminant metabolites with a VIP value > 1.0 and *p*-value < 0.05 were selected using the PLS-DA model. The selected metabolites were tentatively identified by comparison of various analysis data, such as molecular weights, formula, retention time, mass fragment patterns, and mass spectrum of standard compounds, as well as the published references, the chemical dictionary version 7.2 (Chapman and Hall/CRC), in-house library (off-line database in laboratory made by analyzing standards), commercial databases, such as National Institutes of Standards and Technology (NIST) Library (version 2.0, 2011, FairCom, Gaithersburg, MD, USA), and the Human Metabolome Database (HMDB; http://www.hmdb.ca/, (accessed on 4 September 2021)). Significance (*p* < 0.05) was tested by employing the one-way ANOVA and Student’s *t*-test, using predictive analytics software (PASW), Statistics 18 (SPSS Inc., Chicago, IL, USA).

### 2.6. Determination of Total Phenolics, Flavonoids, and Antioxidant Properties

The evaluation of antioxidant activity and bioactive compounds in fermented soy foods were performed using 2,2-azino-bis-(3-ethylbenzothiazoline-6-sulfonic acid) diammonium salt (ABTS), ferric reducing antioxidant power (FRAP), total flavonoid contents (TPC), and total flavonoid contents (TFC) assay. The methods were adopted from those conducted by Jung et al. [14], with modifications. All experiments maintained three replicates of each sample’s extracts.

## 3. Results

### 3.1. Multivariate Statistical Analyses of Different Fermented Soy Products

**Figure 2:**
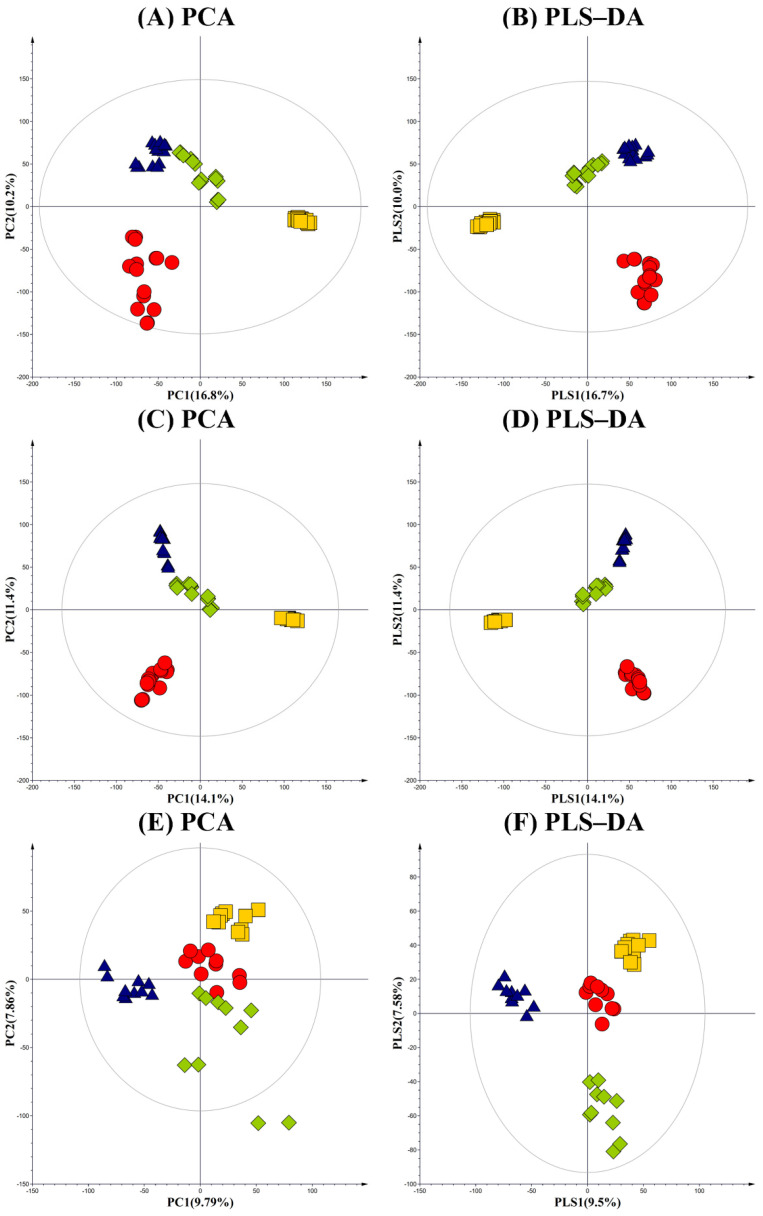
PCA and PLS-DA score plots, derived from the (**A**,**B**) GC-TOF-MS; (**C**,**D**) UHPLC-LTQ-Orbitrap-MS dataset; and (**E**,**F**) HS-SPME/GC-TOF-MS. The datasets are indicated - 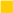; soy, - 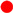; cheonggukjang, 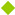; meju, 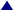; doenjang.

Primary, secondary metabolites and VOCs profiles from cheonggukjang, meju, doenjang, and unfermented soybean were examined using GC–TOF-MS, UHPLC-LTQ-Orbitrap-MS, and HS-SPME/GC-TOF-MS. Variations in metabolite profiles, among raw soybean and fermented soy foods, were examined based on the multivariate analysis of the respective datasets. PCA and PLS-DA models, obtained from GC–TOF-MS analysis, displayed cheonggukjang, meju, and doenjang extracts that were clearly segregated from the unfermented soybean samples across PC1, which suggests their varying metabolite profiles, according to different fermentative bioprocesses. GC-MS datasets for fermented soy foods, meju, and doenjang were clustered separately from cheonggukjang and soybean across PC2, 10.2% (Figure 2A). However, the datasets for each fermented samples were clustered separately from unfermented soybean across PC1, 16.8%. Similar patterns between the datasets for fermented soy foods and soybean were evident in the corresponding PLS-DA plots (Figure 2B). In the PLS-DA plot, the stated satisfaction values of the X and Y variables were 0.324 (R2X) and 0.982 (R2Y), respectively, with a prediction accuracy of 0.970 (Q2).

LC-MS-based PCA and PLS-DA score plots for the secondary metabolite profiles of the fermented soy foods displayed similar patterns as those obtained for GC-MS (Figure 2C,D). Both the PCA and PLS-DA plots highlighted a marked segregation between the fermented soy foods (cheonggukjang, doenjang, and meju) and unfermented soybean across PC1 (14.1%) and PLS1 (14.1%), respectively. LC-MS datasets for meju and doenjang were clustered separately from those for cheonggukjang and soybean across PC2 (11.4%) and PLS2 (11.4%), respectively, in corresponding PCA and PLS-DA plots. In the PLS-DA plot, X and Y variables were 0.352 (R2X), 0.975 (R2Y), and 0.965 (Q2), respectively.

PCA and PLS-DA score plots for the VOCs profiles, based on the HS-SPME/-GC-TOF-MS datasets, indicated distinct patterns, compared to those obtained for primary and secondary metabolites (Figure 2E,F). Most notably, the PCA plot highlighted the clearly segregated VOC datasets obtained for doenjang from rest of the samples (cheonggukjang, meju, and soybean) across PC1 (9.79%), which suggests its distinctive VOCs composition, compared to others. The PLS-DA model displayed similar patterns as PCA model, with statistical variants 0.279 (R2X), 0.979 (R2Y), and 0.908 (Q2).

### 3.2. Significantly Discriminant Metabolites among the Fermented Soy Products

#### 3.2.1. Primary Metabolite Profiling of Soybean and Its Fermented Foods

**Figure 3:**
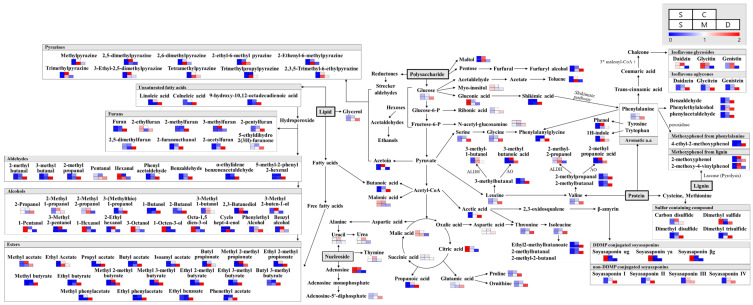
Schematics showing the relative abundance of the significantly discriminant metabolites (primary and secondary metabolites, as well as VOCs), detected comprehensively from the fermented soy foods (cheonggukjang, meju, and doenjang) and unfermented soybean. The metabolites are shown in the form of biosynthetic pathways, adapted from the Kyoto Encyclopedia of Genes and Genomes (KEGG) database, relatively higher in cheonggukjang. Nucleotide compounds were relatively more abundant in cheonggukjang.

Significantly discriminant primary metabolites were selected at a VIP value > 1.0 and *p*-value < 0.05, using the PLS-DA model based on GC-TOF-MS analysis. As shown in Figure 3, the primary metabolites levels were markedly varied among different soy foods made using different fermentation methods. A total of 40 metabolites, including 15 amino acids, 5 carbohydrates, 10 organic acids, 4 lipids, and 4 nucleotides, were selected (Table S3). Notably, all amino acids, as well as most fatty and organic acids, displayed higher relative abundance in doenjang. However, the organic acids associated with the TCA (tricarboxylic acid cycle) cycle, such as citric and malic acid, showed higher relative abundance in unfermented soybean, while succinic and malonic acid were relatively higher in cheonggukjang. Nucleotide compounds were relatively more abundant in cheonggukjang.

#### 3.2.2. Secondary Metabolite Profiling of Soybean and Its Fermented Foods

Totally, 29 significantly discriminant metabolites, including 10 isoflavones, 6 soyasaponins, 7 peptides, and 3 lipids, were selected at a VIP value > 1.0 and *p*-value < 0.05, using the PLS-DA model based on LC-MS datasets (Table S4). Isoflavones and soyasaponins were major metabolites contributing to the observed variance between the metabolite profiles of the different soy products (Figure 3). Notably, the relative levels of β-glucoside derivatives, including daidzin, glycitin, and genistin, were significantly lower in fermented soy products, particularly in doenjang. Glycitin contents were highest in soybean, while daidzin contents were observed more in cheonggukjang. Highest genistin contents were recorded from meju and cheonggukjang. Aglycone, such as daidzein, glycitein, and genistein, contents were recorded highest in doenjang and lowest in soybean. Of the soyasaponins detected in this experiment, all DDMP-conjugated soyasaponins, including soyasaponin αg, βa, and βg, were observed in relatively higher quantities in soybean extracts. Meanwhile, non-DDMP-conjugated soyasaponins, including I, II, III, and IV, were observed in significantly higher quantities in fermented soy products, especially the longer fermented doenjang samples, as compared to the raw and unfermented soybean samples.

#### 3.2.3. Volatile Compounds Profiling of Soybean and Its Fermented Foods

A total of 148 volatile compounds, including 18 organic acids, 20 alcohols, 18 esters, 10 aldehydes, 14 ketones, 8 alkanes/alkenes, 11 aromatic compounds, 4 fatty esters, 11 furans, 5 phenols, 11 pyrazines, and 5 sulfur compounds, were determined as significantly discriminant among different soy products (Table S5). The analysis of flavor-related volatile compounds, using HS-SPME/GC-TOF-MS, indicated that most of the carboxylic acids, aldehydes, esters, furans, sulfur containing compounds, and methoxyphenol displayed higher relative abundance in doenjang, compared to other fermented soy products and soybean. A few of the flavor compounds, including maltol, branched-chain carboxylic acids, and methoxyphenol (from lignin and pyrazine), were relatively higher in cheonggukjang, compared to meju, doenjang, and soybean.

The VOCs profiles detected for doenjang contained higher relative abundance of carboxylic acids, aldehydes, most esters, furans, sulfur containing compounds, and methoxyphenol from phenylalanine. However, the relative levels of maltol and branched-chain carboxylic acids were higher in cheonggukjang. Pyrazine compounds mainly showed the highest content in meju or cheonggukjang, and compounds that were higher in meju also tended to deplete in doenjang, with longer durations of fermentation.

### 3.3. Correlation Assay between Total Phenolics, Flavonoids, Antioxidant Properties, and Metabolites

**Figure 4:**
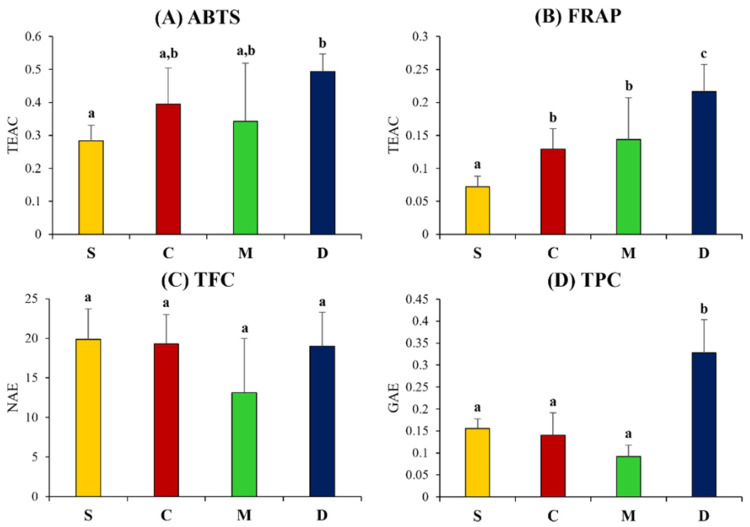
Total flavonoid, phenolic contents, and antioxidant activity tests. (**A**) 2,2-azino-bis-(3-ethylbenzothiazoline-6-sulfonic acid) diammonium salt (ABTS) and (**B**) ferric reducing antioxidant power (FRAP), as well as (**C**) total flavonoid contents (TFC) and (**D**) total phenolic contents (TFC). S, soy; C, cheonggukjang; M, meju; D, doenjang. Values are expressed as the average of three biological replicates (*n* = 3). Bar graphs denoted by the same letter were not significantly different, according to Duncan’s multiple range test (*p*-value < 0.05).

To compare the flavonoid, phenolic contents, and antioxidant activities of soybean and fermented soy products, ABTS, FRAP, total phenolic content (TPC), and total flavonoid content (TFC) were measured (Figure 4). According to the results of the ABTS and FRAP assay, doenjang showed significantly higher antioxidant activity, as compared to cheonggukjang, meju, and unfermented soybean samples. The TPC value was recorded the highest for doenjang among all samples. Notably, the TPC values for fermented (cheonggukjang and meju) and unfermented soybean varied marginally. Unlike other bioactivity assay results, the TFC as showed insignificantly different among fermented soy products and unfermented soybean.

**Figure 5:**
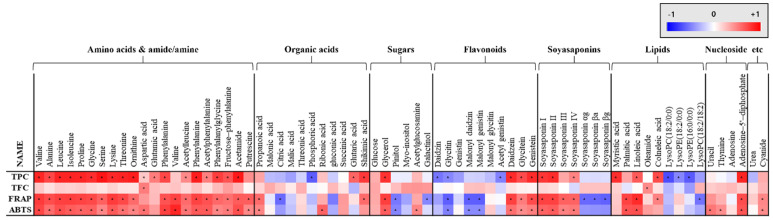
Heat map representation of correlation analysis between relative abundance of significantly discriminant metabolites and antioxidant activity (ABTS and FRAP), total flavonoid content (TFC), and total phenolic content (TPC). Each square indicates Pearson’s correlation coefficient values (r). Red and blue represent positive (0 < r < 1) and negative (−1 < r < 0) correlation, respectively. *; *p*-value less than 0.05, according to Duncan’s multiple range test.

Correlation analysis between the metabolites profiling results and bioactivity assays, such as ABTS, FRAP, TFC, and TPC, were conducted and illustrated in Figure 5. ABTS, FRAP, and TPC showed positive correlations with isoflavone aglycones, such as daidzein, glycitein, and genistein, and negative correlations with β-glucosides, such as daidzin, glycitin, and genistin. In case of soyasaponin, non-DDMP soyasaponins, such as I, II, III, and IV, showed a positive correlation with ABTS, FRAP, and TPC. However, DDMP-conjugated soyasaponins, such as αg, βa, and βg, showed negative correlations with FRAP. Amino acids and dipeptide showed positive correlation with ABTS, FRAP, and TPC values.

## 4. Discussion

Soybean products are widely consumed as fermented foods with varying taste, aroma, and nutritional ingredients, owing to different fermentative bioprocesses. While comparisons between fermented soy foods, manufactured in various ways, have widely studied [9,15,16]. The comprehensive understanding how different fermentative bioprocesses influence end-product metabolomes, including the VOCs, is not completely achieved. Herein, to understand influence of fermentation methods and duration on final fermented soy foods, various fermented soy products (cheonggukjang, meju, and doenjang) with raw materials (soybean) were compared, using metabolite profiling platforms. To do so, multiple hyphenated MS-based platforms GC–TOF-MS, UHPLC-LTQ-Orbitrap-MS, and HS-SPME/GC-TOF-MS were employed. Most primary metabolite contents were significantly higher in doenjang, which can be affected by the long fermentation period (3–6 months), including the brining and aging steps, where both the substrate and microbes contributed to the metabolite pool [17]. Moreover, the fermentative microorganisms of doenjang are usually more diverse and enriched, as compared to those for cheonggukjang or meju, with axenic cultures of *Bacillus* or *Aspergillus* [18,19,20].

Organic acids influence the features of fermented soy products as a flavoring agent by imparting the acidity and sweet flavor. The relatively lower amount of organic acids in doenjang extracts was detected. The organic acids might be consumed as the substrates by microbes during the doenjang fermentation [21] or converted to VOCs [11] (reference [22]). Further, amino acids could be produced from a few organic acid intermediates during the glycolysis and/or TCA cycles [20]. It is known that the higher contents of amino acids are known to influence certain organoleptic traits, including sweetness, bitterness, and savory, in fermented end-products [22]. In this study, the significantly higher levels of most all amino acids was observed in doenjang extracts, as compared to meju, cheonggukjang, and raw soybean.

Considering the secondary metabolite profiles, isoflavones and soyasaponins were the major compounds that varied markedly between fermented soy products (cheonggukjang, meju, and doenjang) and unfermented soybean. Among isoflavones, in raw soybean, significantly higher amounts of isoflavone glucosides (daidzin, glycitin, and genistin) were detected. On the other hand, the isoflavone aglycones (daidzein, glycitein, and genistein) showed comparatively higher contents in doenjang. It is known that the isoflavone glucosides in soybeans are converted to isoflavone aglycones by the β-glucosidases released by the fermentative microorganisms [23]. Moreover, the relative abundance of non-DDMP-conjugated soyasaponins were increased in fermented products, according to the periods of fermentation, i.e., cheonggukjang < meju < doenjang, as described in a previous study [24]. Expectedly, the DDMP-conjugated soyasaponin contents were relatively higher in unfermented soybean. The degradation of DDMP-conjugated soyasaponin could be affected by the unstable characteristic of the DDMP conjugated form and food fermentation [25,26].

Among VOCs, relatively higher amounts of maltol, contributing the sweet flavor [27], were detected in fermented soy products than in raw materials, with a relatively higher abundance in cheonggukjang than doenjang [11]. Generally, maltol is produced by the Maillard reaction of sugars under high temperature and pressure [28]. Hence, we conjecture that the heat applied with rice straw during the soybean pre-processing, prior to cheonggukjang fermentation, might affect higher maltol levels. Additionally, a significantly higher abundances of 3-methylbutanoic acid and 3-methylbutanol were observed in cheonggukjang, which could be produced from 3-methylbutanal by oxidoreductases, according to previous study [29]. Moreover, 2-methoxyphenol and 2-methoxy-4-vinylphenol contents were relatively higher in cheonggukjang. Methoxyphenol is a volatile compound that produces a unique smoky, phenolic odor in fermented foods [30]. These compounds are produced by pyrolysis, followed by laccase-mediated transformation of lignin by Aspergillus [31]. Furthermore, in cheonggukjang and meju, a relatively higher abundance of pyrazines was observed in doenjang, than was in previous studies [32,33], which could be influenced by *B. subtilis* [34], originated from rice straws.

The discrepancy of metabolite contents in different fermented soy foods be caused by the variations in substrate pre-process and fermentation duration. As a result, the total flavonoid and phenolic contents, as well as the antioxidant activities, of various fermented soy products were varied. Doenjang showed significantly higher ABTS, FRAP, and TPC value, as compared to cheonggukjang, meju, and unfermented soybean samples. Similarly, a previous study represented the formation of phenolic compounds, including daidzein, glycitein, and genistein, during fermentation and maturation, which partly affected the high antioxidant activities of fermented products [35].

Further, the statistical correlation analysis was performed to explore particular metabolites, which contribute to bioactivities. Most amino acids, aglycone derivatives of flavonoids, non-DDMP soyasaponins, and fatty acids showed a strong positive correlation with ABTS, FRAP, and TPC. This pattern was in accordance with the previous studies, which have reported on the high antioxidant activities of isoflavone aglycones and non-DDMP soyasaponins in doenjang [16]. Although, it was reported that amino acids have weak antioxidant effects [36], the higher abundance of amino acids in doenjang might enhance its antioxidant activities.

## 5. Conclusions

In order to characterize the food quality, a comprehensive metabolite profiling of different soy fermented products and raw soybeans was carried out, using untargeted metabolite profiling approaches. As a result, in doenjang, the distinctly higher abundance of most organic acids, isoflavone aglycones and non-DDMP soyasaponins, amino acids, and fatty acids might influence its bioactivities. Moreover, the higher VOCs levels in doenjang significantly contributes to the rich taste and aroma of fermented final product. In meju, the fermentative intermediate of doenjang, the relative levels of some pyrazines and alcohols were observed as higher in meju than other soy products, due to the different fermentative processes, such as weaving with rice straws and drying. For cheonggukjang, certain aroma compounds relevant with unique odor, such as pyrazines and methoxyphenols, were more abundant than other soy products, which could be affected by the microflora from rice straws and substrate pre-processing. Based on current results, the observed variations in the metabolomes and volatolomes of soy products might be influenced by varying processing steps, microbial successions, and fermentation duration. This study may help in experimental design, process optimization, and quality control of the soy fermented foods for future research.

## Supplementary Materials

The following are available online at https://www.mdpi.com/article/10.3390/foods10112516/s1, Table S1: Sample information of 5 soy, 5 cheonggukjang, 5 meju, and 5 doenjang. Table S2: The extraction yield of the samples. Table S3: Discriminative metabolites in soy, cheonggukjang, meju, and doenjang, derived from the PLS-DA model of the UHPLC-LTQ-Orbitrap-MS analysis. Table S4: Discriminative metabolites in soy, cheonggukjang, meju, and doenjang, derived from the PLS-DA model of the GC–TOF-MS analysis. Table S5: Discriminative metabolites in soy, cheonggukjang, meju, and doenjang, derived from the PLS-DA model of the HS-SPME/GC-TOF-MS analysis.
