# Supplementary material for: Integrated Metabolomics and Volatolomics for Comparative Evaluation of Fermented Soy Products
Source: Foods. 2021 Oct 20;10(11):2516. doi: 10.3390/foods10112516 (PMC8624193; doi:10.3390/foods10112516)
Supplement: Supplementary file 1 [file foods-10-02516-s001.zip › foods-1414552-supplementary.pdf]

Brand	Location	Sample				Fermentation period			
		S	C	M	D	S	C	M	D
Mygol (1)	Jinan-gun, Jeollabuk-do, Korea	S1	C1	M1	D1	X	-	-	-
Oseak (2)	Yangyang-gun, Gangwon-do, Korea	S2	C2	M2	D2	X	2-3 days	40-50 days	over 2 years
Seoil (3)	Anseong-si, Gyeonggi-do, Korea	S3	C3	M3	D3	X	36 hours	-	over 2 years
Yisak garden (4)	Gongju-si, Chungcheongnam-do, Korea	S4	C4	M4	D4	X	2-3 days	50 days	3 years
Sangchon (5)	Yongin-si, Gyeonggi-do, Korea	S5	C5	M5	D5	X	-	20 days	over 1 year

Table S1 The information of soy(S), cheonggukjang(C), meju(M), doenjang(D) sample.

sample	before extract (mg)	after extract (mg)	extraction yield
S1-1	200.5	17.79	0.08872818
S1-2	200.2	10.34	0.051648352
S1-3	200.41	10.24	0.051095255
S2-1	200.1	11.51	0.057521239
S2-2	200.5	11.23	0.056009975
S2-3	200.07	12.87	0.064327485
S3-1	200.6	12.09	0.060269192
S3-2	200.4	13.03	0.06501996
S3-3	200.6	11	0.054835494
S4-1	200.7	11.84	0.058993523
S4-2	199.2	11.24	0.056425703
S4-3	199.57	10.45	0.05236258
S5-1	199.7	11.75	0.058838257
S5-2	200	13.02	0.0651
S5-3	199.91	12.06	0.060327147
C1-1	199.68	21.69	0.108623798
C1-2	200.41	24.88	0.124145502
C1-3	200.41	21.77	0.108627314
C2-1	200.33	38.65	0.192931663
C2-2	200.07	35.82	0.179037337
C2-3	200.07	48.95	0.244664367
C3-1	199.5	24.73	0.1239599
C3-2	200.6	25	0.124626122
C3-3	200.6	24.2	0.120638086
C4-1	200.06	30.81	0.154003799
C4-2	199.57	31.18	0.156235907

sample	before extract (mg)	after extract (mg)	extraction yield
M1-1	200	20.45	0.10225
M1-2	200.4	22.01	0.109830339
M1-3	200.4	24.5	0.122255489
M2-1	200.7	7.91	0.039412058
M2-2	200.2	7.79	0.038911089
M2-3	200.2	8.01	0.04000999
M3-1	199.4	7.69	0.038565697
M3-2	199.5	18.1	0.090726817
M3-3	199.5	7.99	0.040050125
M4-1	199.4	8.31	0.041675025
M4-2	199.8	8.85	0.044294294
M4-3	199.8	8.29	0.041491491
M5-1	200	16.38	0.0819
M5-2	199.7	14.8	0.074111167
M5-3	199.7	16.83	0.084276415
D1-1	199.97	67.59	0.3380007
D1-2	199.27	62.59	0.314096452
D1-3	199.27	62.11	0.31168766
D2-1	199.38	63.77	0.319841509
D2-2	199.81	62.92	0.314899154
D2-3	199.81	64.08	0.320704669
D3-1	200.21	63.67	0.318016083
D3-2	199.04	62.88	0.315916399
D3-3	199.04	56.7	0.284867363
D4-1	200.13	63.56	0.317593564
D4-2	200.59	64.74	0.322747894

C4-3	199.57	30.09	0.150774164	D4-3	200.59	66.2	0.330026422
C5-1	199.32	21.88	0.109773229	D5-1	200.42	65.48	0.326713901
C5-2	199.91	22.19	0.11099995	D5-2	199.91	64.25	0.321394628
C5-3	199.91	21.79	0.10899905	D5-3	199.91	65.18	0.326046721

Table S2 The Extraction yield of soy(S), cheonggukjang(C), meju(M), doenjang(D) samples

NO.	Tentative identification	UHPLC-LTQ-Orbitrap-MS/MS								
		RT(min)	$[M-H]^-$	$[M+H]^+$	MW	[M-H]- Formula	Error (ppm)	MS fragment ion	VIP	ID
	isoflavone									
1	Daidzin	4.5	415.1043	417.1215	416	C21 H20 O9	12.5	415.10>253.23>223.08>195.08	VIP > 0.7	[1]
2	Glycitin	4.64	445.1154	447.1321	446	C22 H22 O10	2.539	445.12>283.08>268.06	VIP > 0.7	[1]
3	Genistin	5.01	431.0988	433.1166	442	C21 H20 O10	1.74	431.10>268.27>224.05>180.15,194.99,167.19	VIP > 0.7	[1]
4	Malonyl daidzin	5.03	501.0982	503.1225	502	C24 H22 O12	8.144	503.12>417.05,255.25>199.02>181.02	VIP > 0.7	[1]
5	Malonyl genistin	5.42	517.1069	519.1169	518	C24 H22 O13	6.902	519.12>433.08,271.24>215.03>196.99	VIP > 0.7	[1]
6	Malonyl glycitin	5.55	531.1149	533.133	532	C25 H24 O13	0.915	531.11>269.28>240.09,225.02>181.16,175.98	VIP > 0.7	[1]
7	Acetyl genistin	5.78	473.1082	475.1267	474	C23 H22 O11	-1.553	473.11>268.24>239.01>211.03	VIP > 0.7	[1]
8	Daidzein	5.82	253.0502	255.067	254	C15 H10 O4	-1.51	253.05>209.08>165.17	VIP > 0.7	[1]
9	Glycitein	5.95	283.0616	285.078	284	C16 H12 O5	1.248	283.06>268.24>240.03>196.06,184.05,211.07,147.05	VIP > 0.7	[1]
10	Genistein	6.46	269.0452	271.062	270	C15 H10 O5	-1.177	271.06>152.96>66.90,69.01,108.99	VIP > 0.7	[1]
	soyasaponin									
11	Soyasaponin I	7.18	941.5082	943.5333	942	C48 H78 O18	-2.636	941.51>923.59>879.59>733.56,571.60,457.54	VIP > 0.7	[1]
12	Soyasaponin II	7.31	911.498	913.5225	912	C47 H76 O17	-3.274	911.50>615.49>457.41	VIP > 0.7	[1]
14	Soyasaponin III	7.34	795.4521	797.4737	796	C42 H68 O14	-1.634	795.45>615.40>457.44	VIP > 0.7	[1]
15	Soyasaponin IV	7.45	765.4425	767.4638	766	C53 H87 O24	-1.691	765.44>615.48>457.4>437.41,409.46	VIP > 0.7	[1]
16	Soyasaponin αg	7.5	1083.5344	1085.5602	1084	C54 H84 O22	2.406	1083.66>504.55>279.38	VIP > 0.7	[2]
17	Soyasaponin βg	7.57	1067.5377	1069.5653	1068	C54 H84 O21	-1.258	1067.54>967.64>821.60	VIP > 0.7	[2]
	amino acid & peptide									
18	Valine	0.92	116.0724	118.0871	117	C5 H11 N O2	7.154	116.04>99.14,88.13,73.09	VIP > 0.7	[3]
19	Phenylalanine	1.3	164.0722	166.0873	165	C9H11NO2	3.341	164.07>147.24>103.06	VIP > 0.7	[3]
20	Fructose-phenylalanine	1.29	326.1268	328.1414	327	C15 H21 N O7	7.075	326.13>278.11,236.13>260.11,164.10>147.01,112.08	VIP > 0.7	[4]
21	Glycyl-Phenylalanine	2.07	221.0938	223.1095	222	C11H14N2O3	2.869	221.09>164.06>147.04>103.13	VIP > 0.7	[4]
22	Acetylleucine	4.41	172.0985	174.114	173	C8 H15 N O3	8.788	172.10>130.09>112.16,84.21	VIP > 0.7	[3]
23	Acetylphenylalanine	4.76	206.0827	208.0985	207	C11 H13 N O3	7.425	206.08>164.28>146.98>103.05	VIP > 0.7	[3]
	lipid & fatty acid									
24	Linoleic acid	9.2	279.2337	281.2498	280	C18 H32 O2	8.154	279.23>261.21>243.26>130.03	VIP > 0.7	[5]
25	Colneleic acid	9.25	293.213	295.2289	294	C18 H30 O3	7.21	293.21>275.21,245.20,249.24>231.24,161.14,133.16,113.09	VIP > 0.7	[6]
26	Oleamide	10.83	280.2366	282.2805	281	C18 H36 N O	-0.663	(+) 282.28>265.43>247.19>149.08,163.10,135.04	VIP > 0.7	[7]

27	LysoPC(18:2/0:0)	8.52	564.3299	520.3425	519	C26 H50 N O7 P	5.255	(+)520.34>502.38,184.25>443.20,86.06>307.21,162.95,71.12	VIP < 0.7	[8]
28	LysoPE(18:2/0:0)	8.44	476.278	478.2962	477	C23 H44 N O7 P	-0.551	476.28>279.47>261.28>243.21	VIP < 0.7	[8]
29	LysoPE(16:0/0:0)	8.72	452.2786	454.2962	453	C21 H44 N O7 P	0.746	452.28>378.31,255.41,152.98>237.22,140.11>183.25,79.02	VIP < 0.7	[8]
30	LysoPC(18:2/18:2)	11.46	-	782.5725	781	C44 H80 N O8 P	3.921	782.57>520.30>502.29>443.17	VIP < 0.7	[8]
	etc									
31	Trehalose	1.22	341.1091	343.1258	342	C12 H22 O11	0.573	341.11>179.03>143.00	VIP > 0.7	[9]
32	Galactinol	0.89	341.1095	343.0932	342	C12 H22 O11	1.657	341.11>179.03,161.02>143.00>115.10	VIP > 0.7	[10]
33	Shikimic acid	0.88	175.059	173.0461	174	C7 H10 O5	-6.048	173.01>129.01>85.05	VIP > 0.7	[11]

Table S3 Discriminative metabolites in soy, choenggukjang, meju, doenjang derived from the PLS-DA model of the UHPLC-LTQ-orbitrap-MS analysis.
RT; Retention time

No.	Ret(min)[a]	Metabolites[b]	Unique Mass (m/z)	MS Fragment pattern (m/z)	TMS	Identification[c]
Amino acids & amine, amide						
1	5.17	Valine	72	72,55,75,73,74,146,56,130,103,156	1	Lib/STD
2	5.31	Alanine	116	116,73,147,117,59,74,75,100,103,118	2	Lib/STD
3	5.88	Leucine	86	86,75,73,74,87,103,146,61,188,130	1	Lib/STD
4	6.47	Acetamide	110	73,110,228,134,77,184,69,75,58,74	1	Lib/MS
5	7.27	Isoleucine	158	73,158,100,218,147,159,74,59,75,86	2	Lib/STD
6	7.32	Proline	142	142,73,70,143,147,74,59,66,144,72	2	Lib/STD
7	7.40	Glycine	174	73,174,86,147,100,59,133,175,248,74	3	Lib/STD
8	7.90	Serine	204	73,100,188,147,59,204,114,133,218,74	3	Lib/STD
9	8.15	Threonine	219	73,117,59,101,219,218,147,100,75,74	3	Lib/STD
10	8.46	Aspartic acid	160	73,160,75,130,117,116,74,147,70,100	2	Lib/STD
11	9.55	Phenylalanine	120	120,146,73,75,130,91,103,74,77,121	1	Lib/STD
12	10.07	Glutamic acid	128	73,128,246,84,75,147,156,56,100,74	3	Lib/STD
13	10.98	Putrescine	174	174,73,86,59,175,100,176,74,130,214	4	Lib/MS
14	11.55	Ornithine	142	73,142,174,147,86,74,59,100,143,75	4	Lib/MS
15	12.25	Lysine	156	73,156,174,128,59,86,100,74,147,230	4	Lib/STD
Carbohydrates						
16	7.08	Glycerol	218	73,147,117,103,133,218,205,148,129,59	3	Lib/STD
17	11.75	Pinitol	217	73,217,147,260,191,133,159,318,207,129	5	Lib/MS
18	12.20	Glucose	160	147,160,205,217,157,129,319,229,148,189	5	Lib/STD
19	12.58	Myo-inositol	318	73,147,217,45,191,129,103,74,133,75	6	Lib/STD
20	13.39	N-Acetyl-glucosamine	87	73,147,129,87,202,117,75,103,205,173	4	Lib/STD
Organic acids						
21	5.75	Propanoic acid	177	147,73,75,177,148,219,55,101,59,149	2	Lib/MS
22	6.39	Malonic acid	147	147,73,75,148,66,149,74,72,59,233	2	Lib/STD
23	7.44	Succinic acid	247	147,73,75,148,55,56,247,149,129,172	2	Lib/MS
24	9.02	Malic acid	147	73,147,75,55,133,74,101,233,148,59	3	Lib/STD
25	9.65	Threonic acid	292	73,147,117,103,102,130,292,74,220,55	4	Lib/MS
26	9.71	Glutaric acid	129	129,73,147,75,85,247,157,203,133,149	3	Lib/MS
27	11.19	Phosphoric acid	299	73,299,147,75,101,103,357,133,59,129	4	Lib/STD
28	11.36	Ribonic acid	292	73,147,103,217,292,74,117,133,189,148	5	Lib/MS

29	11.6	Citric acid	211	73,147,211,183,273,75,67,375,74,149	4	Lib/STD
30	12.89	gluconic acid	333	73,147,103,217,205,292,129,117,74,133	5	Lib/STD
Lipids						
31	11.68	Myristic acid	132	75,117,132,129,55,145,285,59,76,95	1	Lib/MS
32	12.98	Palmitic acid	117	73,75,117,43,132,41,129,55,313,145	1	Lib/MS
33	14.02	Linoleic acid	337	75,73,67,81,129,55,337,95,117,79	1	Lib/STD
34	15.15	Oleamide	128	75,73,131,55,116,128,144,54,74,69	1	Lib/STD
Nucleotides						
35	7.12	Adenosine-5'-diphosphate	133	93,133,299,193,74,211,207,135,191,75	3	Lib/STD
36	7.72	Uracil	99	99,241,255,126,113,256,100,73,242,131	2	Lib/STD
37	8.29	Thymine	255	255,113,73,147,59,270,120,256,140,100	2	Lib/MS
38	16.41	Adenosine	230	73,45,236,230,103,74,75,147,192,245	4	Lib/STD
Etc.						
39	6.78	Urea	171	147,171,73,189,99,74,148,66,100,75	2	Lib/MS
40	15.23	Cyanide	84	84,73,85,86,74,99,75,100,87	1	Lib/MS

Table S4 Discriminative metabolites in soy, choenggukjang, meju, doenjang derived from the PLS-DA model of the GC–TOF-MS analysis. [a] Retention time; [b] Metabolites were selected and identified based on VIP > 0.7 and p-value <0.05 in both VIP1 and VIP2 by PLS-DA; [c] Identification: STD, mass fragment pattern was consistent with those of commercial standard compounds; MS, mass fragment pattern was consistent with those of NIST and in-house libraries

No.	Sample	Ret(min)	Metabolites	Unique Mass (m/z)	MS Fragment pattern (m/z)	CAS	Formula	Weight	Identification
Acids									
1	all	1.235	Carbamic acid	44	44,40,147,45,66,73,148,46	1111-78-0	CH6N2O2	78	Lib/MS
2	C	19.0017	Acetic acid	60	45,43,60,44,42,41,40,61,46,47	64-19-7	C2H4O2	60	Lib/STD
3	D	21.23	Propanoic acid	74	44,45,74,73,57,56,55,46,42,43	1979-09-04	C3H6O2	74	Lib/STD
4	C,M,D	21.8033	2-methylpropanoic acid	73	43,41,73,44,39,42,45,88,40,55	79-31-2	C4H8O2	88	Lib/MS
5	M,D	22.9883	Butanoic acid	60	60,44,73,39,45,42,43,55,38,40	107-92-6	C4H8O2	88	Lib/STD
6	C,M	23.7017	2-Methylbutanoic acid	74	74,41,57,39,60,45,87,44,73,43	116-53-0	C5H10O2	102	Lib/MS
7	C,M	23.7133	3-Methylbutanoic acid	60	60,43,41,45,42,39,87,61,44,69	503-74-2	C5H10O2	102	Lib/MS
8	C,D	25.7533	3-Methyl-2-butenoic acid	100	100,39,82,85,55,44,83,43,41,54	541-47-9	C5H8O2	100	Lib/MS
9	C,D	24.8617	Pentanoic acid	60	60,73,41,39,42,45,56,55,61,40	109-52-4	C5H10O2	102	Lib/STD
10	C,D	25.8383	4-Methylpentanoic acid	74	57,41,73,74,43,55,60,44,39,42	646-07-1	C6H12O2	116	Lib/MS
11	M,D	26.4467	Hexanoic acid	60	60,73,41,42,43,45,39,44,55,61	142-62-1	C6H12O2	116	Lib/MS
12	C,D	27.545	4-Methylhexanoic acid	73	73,55,41,43,71,74,60,44,39,83	52745-93-4	C7H14O2	130	Lib/MS
13	C,D	29.2583	Octanoic acid	60	60,73,43,41,55,44,39,61,101,42	124-07-2	C8H16O2	144	Lib/MS
14	C,M,D	30.5183	Nonanoic acid	60	60,73,41,57,43,55,44,115,39,61	112-05-0	C9H18O2	158	Lib/MS
Alcohols									
15	S,C,M	2.16	2-Methyl-2-propanol	59	59,41,43,39,57,56,42,60,55,40	75-65-0	C4H10O	74	Lib/MS
16	S,D	2.40833	2-Propanol	45	45,43,41,39,44,42,59,46,38,40	67-63-0	C3H8O	60	Lib/MS
17	D	3.75167	2-Butanol	45	45,41,59,43,44,39,55,56,42,57	14898-79-4	C4H10O	74	Lib/MS
18	C,D	4.06833	1-Propanol	60	42,59,41,60,39,43,40,38,57,58	71-23-8	C3H8O	60	Lib/MS
19	D	5.85	2-Methyl-1-propanol	42	41,43,42,39,56,40,38,74,55,37	78-83-1	C4H10O	74	Lib/MS
20	D	7.71	1-Butanol	56	41,56,43,42,39,55,44,40,57,45	71-36-3	C4H10O	74	Lib/MS
21	C	10.1467	3-Methyl-1-butanol	55	55,42,41,43,39,70,45,44,57,46	123-51-3	C5H12O	88	Lib/MS
22	C	9.745	3-Methyl-2-pentanol	45	45,41,44,56,43,39,55,57,69,42	565-60-6	C6H14O	102	Lib/MS
23	S,M	11.7717	1-Pentanol	42	44,39,77,43,46,38,338,57,56,55	71-41-0	C5H12O	88	Lib/MS
24	C	14.4233	3-Methyl-2-buten-1-ol	71	71,39,41,53,67,68,86,65,55,54	556-82-1	C5H10O	86	Lib/MS

25	S,M	15.5117	1-Hexanol	56	56,41,55,43,42,39,69,57,53,40	111-27-3	C6H14O	102	Lib/MS
26	S	16.9533	3-Octanol	83	59,55,41,83,43,57,58,101,42,39	589-98-0	C8H18O	130	Lib/MS
27	all	18.8333	1-Octen-3-ol	57	57,43,41,72,55,39,58,42,54,67	3391-86-4	C8H16O	128	Lib/MS
28	S	19.81	Octa-1,5-dien-3-ol	57	57,55,70,39,53,58,68,79,69,41	50306-18-8	C8H14O	126	Lib/MS
29	C,M	19.93	2-Ethylhexanol	57	57,41,43,55,56,70,83,39,42,69	104-76-7	C8H18O	130	Lib/MS
30	C,M	22	2,3-Butanediol	45	45,43,44,57,47,42,46,41,55,75	513-85-9	C4H10O2	90	Lib/MS
31	M	22.5033	Cyclohept-4-enol	79	79,67,39,94,41,77,53,58,55,66	38607-27-1	C7H12O	112	Lib/MS
32	D	24.4467	3-(Methylthio)-1-propanol	61	61,57,73,41,47,59,46,58,60,106	505-10-2	C4H10OS	106	Lib/MS
33	C,M,D	26.8567	Benzyl alcohol	108	79,108,77,107,51,39,50,91,78,105	100-51-6	C7H8O	108	Lib/MS
34	C,M,D	27.3183	Phenylethyl Alcohol	91	91,92,65,122,39,51,77,63,78,50	1960-12-08	C8H10O	122	Lib/MS
35	C,M,D	28.0783	Maltol	126	126,71,43,55,42,97,44,69,53,54	118-71-8	C6H6O3	126	Lib/MS
Esters									
36	C	1.25667	Methyl formate	60	60,77,131,61,52,36,62,58,105,259	107-31-3	C2H4O2	60	Lib/MS
37	S,C	1.42	Vinyl formate	43	44,40,43,147,42,59,41,73,45,66	692-45-5	C3H4O2	72	Lib/MS
38	C	1.53833	Butyl propionate	56	57,56,75,39,58	590-01-2	C7H14O2	130	Lib/MS
39	C,M,D	1.76833	Methyl acetate	74	43,74,42,59,44,41,45,75,58,73	79-20-9	C3H6O2	74	Lib/MS
40	all	2.08167	Ethyl Acetate	73	43,45,61,70,42,44,73,88,60,41	141-78-6	C4H8O2	88	Lib/MS
41	C,D	2.36	Methyl-2-methylpropanoate	87	43,71,41,44,59,87,42,39,55,102	547-63-7	C5H10O2	102	Lib/MS
42	C,D	2.83167	Ethyl 2-methylpropionate	71	43,71,41,45,88,42,44,73,39,116	97-62-1	C6H12O2	116	Lib/MS
43	C,D	2.945	Propyl acetate	61	43,61,73,42,41,44,39,59,40,57,72	109-60-4	C5H10O2	102	Lib/MS
44	D	3.095	Methyl butyrate	74	74,43,71,59,41,42,87,39,55,45	623-42-7	C5H10O2	102	Lib/MS
45	C,D	3.465	Methyl 2-methylbutyrate	88	41,40,88,57,39,59,85,38,56,101	868-57-5	C6H12O2	116	Lib/MS
46	C,D	3.655	Methyl 3-methylbutanoate	74	74,43,59,41,85,39,57,42,101,69	556-24-1	C6H12O2	116	Lib/MS
47	D	4.00333	Ethyl butyrate	71	71,43,88,41,60,73,45,42,91,39	105-54-4	C6H12O2	116	Lib/MS
48	C,D	4.36833	Ethyl 2-methyl butanoate	57	57,41,102,85,74,87,56,45,39,55	7452-79-1	C7H14O2	130	Lib/MS
49	C,D	4.78333	Ethyl 3-methyl butanoate	88	88,60,85,41,57,43,61,70,39,44	108-64-5	C7H14O2	130	Lib/MS
50	D	4.895	Butyl acetate	43	43,56,41,73,61,39,42,55,57,58	123-86-4	C6H12O2	116	Lib/MS
51	D	6.52833	Isoamyl acetate	61	43,55,70,41,42,44,61,39,73,87	123-92-2	C7H14O2	130	Lib/MS
52	M	9.04	Methyl hexanoate	74	74,43,44,59,87,41,42,39,55,99	106-70-7	C7H14O2	130	Lib/MS

53	D	11.525	Butyl 3-methyl butanoate	85	41,85,56,57,103,60,39,43,44,87	109-19-3	C9H18O2	158	Lib/MS
54	D	10.8067	Butyl 2-methylbutanoate	85	57,41,103,85,56,74,44,39,43,55	15706-73-7	C9H18O2	158	Lib/MS
55	C	16.6317	Methyl octanoate	74	74,43,87,41,55,59,57,58,115,39	111-11-5	C9H18O2	158	Lib/MS
56	C	18.1783	Ethyl octanoate	88	88,60,57,61,70,73,41,101,43,55	106-32-1	C10H20O2	172	Lib/MS
57	D	23.515	Ethyl benzoate	105	105,77,122,51,150,106,50,78,45,76	93-89-0	C9H10O2	150	Lib/MS
58	C,D	25.135	Methyl phenylacetate	91	91,150,65,44,39,59,63,92,89,90	101-41-7	C9H10O2	150	Lib/MS
59	D	25.525	Ethyl phenylacetate	91	91,65,164,92,39,89,63,51,90,105	101-97-3	C10H12O2	164	Lib/MS
60	D	25.9567	Phenethyl acetate	104	43,104,44,91,39,105,69,51,78,41	103-45-7	C10H12O2	164	Lib/MS
Aldehydes									
61	C,M,D	1.70167	2-Methylpropanal	72	41,72,39,38,57,37,44,42,73,70	78-84-2	C4H8O	72	Lib/MS
62	C,M,D	2.27	2-Methylbutanal	57	41,57,58,39,43,55,44,42,56,75	96-17-3	C5H10O	86	Lib/MS
63	all	2.31167	3-Methylbutanal	41	44,41,58,43,39,42,71,57,45,38	590-86-3	C5H10O	86	Lib/MS
64	D	2.99833	Pentanal	58	44,41,58,57,39,43,45,42,55,38	110-62-3	C5H10O	86	Lib/MS
65	S,M,D	5.12	Hexanal	57	44,41,56,57,43,39,55,42,45,72	66-25-1	C6H12O	100	Lib/MS
66	C,M,D	20.5467	Benzaldehyde	106	77,106,105,51,50,78,52,107,74,39	100-52-7	C7H6O	106	Lib/MS
67	D	23.1433	Benzeneacetaldehyde	91	91,120,65,92,50,62,41,51,64,89	122-78-1	C8H8O	120	Lib/MS
68	D	27.595	α-Ethylidenebenzeneacetaldehyde	115	115,117,146,91,116,39,51,63,78,118	4411-89-6	C10H10O	146	Lib/MS
69	D	29.4233	5-Methyl-2-phenyl-2-hexenal	117	117,104,43,115,91,41,116,103,39,188	21834-92-4	C13H16O	188	Lib/MS
Ketones									
70	all	1.71833	Acetone	58	43,58,42,39,38,41,37,59,57	67-64-1	C3H6O	58	Lib/MS
71	C,M,D	2.17667	2-Butanone	72	43,72,57,42,44,39,41,53,45,73	78-93-3	C4H8O	72	Lib/MS
72	S,C,D	2.96667	2-Pentanone	43	45,46,43,49,84,86,42,51,44,47	107-87-9	C5H10O	86	Lib/MS
73	C,M	3.03667	2,3-Butanedione	43	43,86,42,44,41,39,75,49,87,45	431-03-8	C4H6O2	86	Lib/MS
74	C,M	8.82333	2-Heptanone	58	43,58,59,41,39,42,71,72,55,57	110-43-0	C7H14O	114	Lib/MS
75	M,D	12.785	2-Octanone	58	43,58,41,59,71,39,85,55,42,57	111-13-7	C8H16O	128	Lib/MS
76	C,M	12.8433	Acetoin	45	45,43,42,44,88,41,46,73,55,39	513-86-0	C4H8O2	88	Lib/MS
77	S	13.4317	1-Octen-3-one	70	55,70,43,41,97,39,83,42,71,56	4312-99-6	C8H14O	126	Lib/MS
78	M	14.54	6-Octen-2-one	43	43,41,68,55,67,39,97,58,69,71	74810-53-0	C8H14O	126	Lib/MS
79	D	22.2717	2-Undecanone	58	58,43,71,59,57,51,69,55,77,91	112-12-9	C11H22O	170	Lib/MS

80	M	23.2267	Acetophenone	105	105,77,51,120,43,50,78,106,74,38	98-86-2	C8H8O	120	Lib/MS
81	M,D	24.5783	Phenylacetone	91	43,91,92,65,134,39,63,51,89,41	103-79-7	C9H10O	134	Lib/MS
Alkanes & Alkenes									
82	M	1.25667	Isopentane	56	41,39,56,42,43,57,55,50,53,51	78-78-4	C5H12	72	Lib/MS
83	C,M,D	1.39	2-Methoxy-2-methylpropane	73	73,41,43,57,147,39,56,45,55,42	1634-04-4	C5H12O	88	Lib/MS
84	C	1.42833	2-ethoxy-2-methylpropane	87	59,57,87,147,60,88,148,131,103	637-92-3	C6H14O	102	Lib/MS
85	D	1.63	Octane	41	43,41,57,39,42,56,85,71,55,58	111-65-9	C8H18	114	Lib/MS
86	M	20.0767	1-Nitro-hexane	55	43,41,55,39,42,57,62,59,56,45	646-14-0	C6H13NO2	131	Lib/MS
87	C	1.36	2-Methyl-1-pentene	84	41,56,39,55,42,69,84,53,67,43	763-29-1	C6H12	84	Lib/MS
88	M,D	2.025	2,4-Dimethyl-1-heptene	55	43,70,41,55,56,57,39,42,69,71	19549-87-2	C9H18	126	Lib/MS
Aromatic compounds									
89	C,M	4.005	Toluene	65	91,92,65,39,63,51,45,50,89,93	108-88-3	C7H8	92	Lib/MS
90	M	6.275	Ethylbenzene	91	91,106,51,65,77,39,78,92,50,63	100-41-4	C8H10	106	Lib/MS
91	D	22.3467	Benzonitrile	103	103,76,50,51,75,104,77,52,39,38	100-47-0	C7H5N	103	Lib/MS
92	D	22.69	Methyl benzoate	105	105,77,136,51,50,106,81,78,39,76	93-58-3	C8H8O2	136	Lib/MS
93	M,D	23.7533	1-ethenyl-4-methoxybenzene	134	134,119,91,65,38,51,63,77,135,75	637-69-4	C9H10O	134	Lib/MS
94	D	28.035	1,2,3-Trimethoxybenzene	168	44,168,153,93,110,39,95,125,65,41	634-36-6	C9H12O3	168	Lib/MS
95	M,D	28.1583	2-Acetylpyrrole	94	94,109,66,39,43,38,40,53,37,110	1072-83-9	C6H7NO	109	Lib/MS
96	M	28.9783	4-ethenyl-1,2-dimethoxybenzene	164	164,77,91,149,103,78,121,65,51,50	6380-23-0	C10H12O2	164	Lib/MS
97	M	33.8333	1H-Indole	117	117,90,89,63,116,118,62,39,64,38	120-72-9	C8H7N	117	Lib/MS
Furans									
98	D	1.64167	Furan	68	39,68,38,37,42,147,69,36,45,67	110-00-9	C4H4O	68	Lib/MS
99	D	1.975	2-Methylfuran	82	82,81,53,39,51,50,43,54,38,41	534-22-5	C5H6O	82	Lib/MS
100	C,D	2.14	3-Methylfuran	82	82,81,53,39,50,54,51,38,83,37	930-27-8	C5H6O	82	Lib/MS
101	D	2.67	2,5-Dimethylfuran	96	96,95,53,81,43,50,39,67,41,97	625-86-5	C6H8O	96	Lib/MS
102	S,C	2.67333	2-Ethylfuran	81	45,46,49,43,84,86,42,51,44,47	3208-16-0	C6H8O	96	Lib/MS
103	C,M,D	10.6683	2-Pentylfuran	81	81,82,53,138,41,39,95,94,67,68	3777-69-3	C9H14O	138	Lib/MS
104	D	20.2567	2-Acetylfuran	110	95,110,39,43,67,96,41,38,37,51	1192-62-7	C6H6O2	110	Lib/MS
105	C,M,D	23.5983	2-Furanmethanol	98	98,41,39,42,97,81,53,69,70,38	98-00-0	C5H6O2	98	Lib/MS

106	M,D	24.135	5-Ethyldihydro-2(3H)-furanone	85	85,41,57,56,42,39,55,86,40,43	695-06-7	C6H10O2	114	Lib/MS
Phenols									
107	C,M,D	26.64	2-Methoxyphenol	109	109,124,81,53,39,51,52,110,50,63	1990-05-01	C7H8O2	124	Lib/MS
108	C,M,D	28.6	Phenol	94	94,66,65,39,40,55,38,47,63,95	108-95-2	C6H6O	94	Lib/MS
109	D	28.8883	4-Ethyl-2-methoxyphenol	137	137,152,122,39,91,77,138,51,94,65	2785-89-9	C9H12O2	152	Lib/MS
110	D	30.6517	4-Ethylphenol	107	107,122,77,39,108,51,53,91,65,63	123-07-9	C8H10O	122	Lib/MS
111	C,M	30.8983	2-Methoxy-4-vinylphenol	135	135,150,77,107,39,51,79,78,151,50	7786-61-0	C9H10O2	150	Lib/MS
Pyrazines									
112	M	12.07	methyl pyrazine	94	94,67,40,39,53,42,41,38,52,37	109-08-0	C5H6N2	94	Lib/MS
113	C,M,D	14.0883	2,5-Dimethylpyrazine	108	42,108,39,40,81,38,41,52,109,37	123-32-0	C6H8N2	108	Lib/MS
114	C,M,D	14.31	2,6-Dimethylpyrazine	108	108,42,40,39,41,38,81,66,52,109	108-50-9	C6H8N2	108	Lib/MS
115	C,M,D	14.9433	2,3-Dimethylpyrazine	108	67,108,40,42,41,39,52,51,38,109	5910-89-4	C6H8N2	108	Lib/MS
116	C,M,D	16.2833	2-Ethyl-6-methylpyrazine	121	121,122,44,39,40,94,56,66,42,53	13925-03-6	C7H10N2	122	Lib/MS
117	C,M,D	16.9433	Trimethylpyrazine	122	42,122,39,81,40,54,53,41,52,38	14667-55-1	C7H10N2	122	Lib/MS
118	C,M,D	18.3167	3-Ethyl-2,5-dimethylpyrazine	135	135,136,42,39,56,108,107,40,41,53	13360-65-1	C8H12N2	136	Lib/MS
119	C,M,D	19.2483	Tetramethylpyrazine	136	54,42,136,39,53,52,51,41,55,60	1124-11-4	C8H12N2	136	Lib/MS
120	M	19.695	2-Ethenyl-6-methylpyrazine	120	120,52,39,119,54,51,94,50,121,42	13925-09-2	C7H8N2	120	Lib/MS
121	C,M,D	20.2983	2,3,5-Trimethyl-6-ethylpyrazine	149	149,150,42,53,39,54,67,122,41,121	17398-16-2	C9H14N2	150	Lib/MS
122	M,D	21.8617	trimethyl propyl pyrazine	136	136,149,135,53,137,54,52,51,94,42	92233-82-4	C10H16N2	164	Lib/MS
Sulfur compounds									
123	C	1.47667	Carbon disulfide	76	76,78,38,77,47,39,87,117,55,64	75-15-0	CS2	76	Lib/MS
124	D	1.51	Dimethyl sulfide	62	62,47,45,44,46,35,40,61,43,48	75-18-3	C2H6S	62	Lib/MS
125	D	4.81	Dimethyl disulfide	94	94,45,79,46,47,48,61,96,64,44	624-92-0	C2H6S2	94	Lib/MS
126	D	5.30667	2-Methylthiophene	97	97,98,53,99,58,69,50,71,49,51	554-14-3	C5H6S	98	Lib/MS
127	D	15.805	Dimethyl trisulfide	126	126,45,79,44,47,46,64,80,111,128	3658-80-8	C2H6S3	126	Lib/MS
Etc.									
128	S,M	1.33833	Ethyl ether	59	44,147,40,45,59,73,66,148,74,43	60-29-7	C4H10O	74	Lib/MS
129	C,M,D	1.38333	Methanethiol	48	40,44,147,47,48,45,73,66,41,148	0-00-0	C2H4S	60	Lib/MS
130	all	2.46167	Dichloromethane	84	49,84,86,51,47,48,41,88,35,42	1975-09-02	CH2Cl2	84	Lib/STD

131	S,C,M	3.47	Trimethylborane	41	41,40,39,38,42	593-90-8	C3H9B	56	Lib/MS
132	M	3.71667	Chloroform	83	83,85,47,48,87,49,35,44,40,75	67-66-3	CHCl3	118	Lib/MS
133	M,D	6.69333	3-Methylbutanenitrile	41	41,43,39,40,42,38,68,37,55,52	625-28-5	C5H9N	83	Lib/MS
134	C,M	9.40333	Trimethyloxazole	111	43,111,55,42,68,70,41,69,82,110	20662-84-4	C6H9NO	111	Lib/MS
135	D	24.7483	N-(2-Methylpropyl)acetamide	72	43,72,60,100,58,73,41,115,42,56	1540-94-9	C6H13NO	115	Lib/MS
136	S,C,M	25.31	Methoxyphenyloxime	77	133,151,135,68,77,134,45,75,152,89	0-00-0	C8H9NO2	151	Lib/MS
137	D	27.56	Benzyl nitrile	117	117,90,116,89,51,39,63,91,50,77	140-29-4	C8H7N	117	Lib/MS

Table S5 Discriminative metabolites in soy, choenggukjang, meju, doenjang derived from the PLS-DA model of the SPME-GC/MS analysis. [a] Retention time; [b] Metabolites were selected and identified based on VIP > 0.7 and p-value <0.05 in both VIP1 and VIP2 by PLS-DA; [c] Identification: STD, mass fragment pattern was consistent with those of commercial standard compounds; MS, mass fragment pattern was consistent with those of NIST and in-house libraries (S; soy, C; cheonggukjang, M; meju, D; doenjang)
